# Glucagon-Like Peptide-1 (GLP-1) during Ramadan: Narrative Review of the Published Literature

**DOI:** 10.1155/2023/8626081

**Published:** 2023-12-26

**Authors:** Khalid Mohammed Alayed

**Affiliations:** Department of Medicine, College of Medicine, King Saud University, Riyadh, Saudi Arabia

## Abstract

Ramadan fasting, a religious practice observed by Muslims worldwide, involves abstaining from eating, drinking, smoking, and using oral medications from dawn to dusk during the ninth lunar month. Studies have demonstrated that fasting during Ramadan has been shown to increase HDL cholesterol, leptin, adiponectin, and insulin sensitivity, as well as lower several hemostatic risk factors for cardiovascular diseases. Additionally, it may result in a drop in blood sugar levels, especially in diabetics who are also on blood sugar-lowering medicine. Hypoglycemia, characterized by low blood sugar levels, could also result from fasting during Ramadan. The GLP-1 (glucagon-like peptide-1) hormone plays a significant role in regulating glucose metabolism and insulin secretion, and Ramadan fasting can affect its production and release in the gut. Research contributes to our understanding of the utilization of GL-1 medications during Ramadan among patients, broadening therapy alternatives and offering insightful information for well-informed decision-making. Therefore, this narrative review aims to explore the current evidence that studies the safety and efficacy of GLP-1 agonists during Ramadan for nondiabetic and diabetic patients to ensure healthy fasting during Ramadan.

## 1. Introduction

The Islamic religious observance of Ramadan, one of the Five Pillars of Islam, occurs during the ninth lunar month [1]. During this time, Muslims worldwide engage in a prescribed fast from dawn until dusk, abstaining from all food, drink, and oral medication consumption including diabetic patients [2].

The impact of Ramadan fasting on blood glucose levels among nondiabetic individuals has been the subject of conflicting findings in the literature [3]. Some studies have reported a decrease in blood glucose levels during the fast, while others have documented an increase. During Ramadan fasting, adipokines, such as adiponectin, known for enhancing insulin sensitivity, may collaborate with GLP-1 to improve glucose control [4]. The ratio of adiponectin to leptin and other adipokines might play a role in regulating metabolic responses during fasting, although specific interactions require further research for a comprehensive understanding [5].

Additionally, it has been linked to improvements in several hemostatic risk factors for cardiovascular diseases, including decreased plasma levels of LDL cholesterol and triglycerides [6]. Ramadan fasting is linked to lower plasma levels of homocysteine, D-dimer, C-reactive protein (CRP), IL-6, and fibrinogen in nondiabetics [7]. An investigation found that, although nondiabetic participants experienced a decrease in blood glucose levels during the fast, a subsequent increase was observed after breaking the fast, potentially due to excessive food intake or the consumption of high-calorie foods during the evening meal (Iftar) [8]. Conversely, a study published in the Journal of Diabetes and Metabolic Disorders in 2014 found that Ramadan fasting was associated with increased blood glucose levels among nondiabetic individuals, which may be attributed to alterations in circadian rhythms and the timing of meals [4]. On the other hand, individuals with diabetes who are receiving medication to lower their blood sugar levels have been shown to experience a decrease in blood glucose levels during Ramadan fasting due to the temporary cessation of medication intake [9].

GLP-1 is a hormone known for its roles in insulin stimulation and glucagon reduction [10] and plays a vital role in blood sugar control. This significance becomes particularly important during fasting in diabetic individuals, such as during Ramadan [11]. It helps in managing glucose levels during the fasting period [11], making it a critical component in diabetes management. The hormone is produced in the gut and released into the bloodstream in response to food, particularly fat, protein hydrolysates, and glucose [12]. The use of glucagon-like peptide (GLP) agonists as an effective treatment option for obesity and type 2 diabetes mellitus (T2DM) is supported by high-quality data [13]. Numerous studies have evaluated the safety and efficacy of GLP-1 receptor agonists in diabetic patients during Ramadan [14]. Fasting is linked to higher levels of leptin, adiponectin, and HDL cholesterol in nondiabetics [15]. GLP-1 receptor agonists are related to significant body weight reduction and improvements in glucose metabolism, helping diabetic patients who are fasting by regulating blood glucose levels by stimulating insulin secretion and reducing glucagon production [16].

Hypoglycemia, a disorder characterized by low blood glucose levels [17], has been identified as a potential consequence of Ramadan fasting [18]. Decreased meal intake during fasting has been established as a risk factor for developing hypoglycemia [19]. When the body's need for glucose surpasses the supply available, there is an increased risk of hypoglycemia, especially in individuals with diabetes who are taking insulin or other diabetes medications [20]. Reduced food intake during fasting has been recognized as a contributing factor to hypoglycemia [21], and this risk is higher in people with diabetes who rely on insulin or antidiabetic medications to manage their condition [22]. To avoid unwanted gastrointestinal discomfort and reduce the risk of hypoglycemia, dose titration must be performed at least four weeks before Ramadan [23], as the median time for the start of hypoglycemia is typically five days after GLP administration [24]. A study reported that patients with type 1 diabetes who took oral antidiabetes drugs and fasted during Ramadan had a 4.7-fold higher risk of severe hypoglycemia, increasing from 3 to 14 occurrences per 100 people per month to 7.5 events per 100 people per month [25]. In comparison, patients with type 2 diabetes had a 0.4-fold increase in the risk of severe hypoglycemia [25]). Sulfonylurea use has increased the risk of hypoglycemia in diabetes patients [26]. Patients who underwent changes in their oral hypoglycemic medication or insulin dosage or reported significant changes in their lifestyle were also found to have a higher likelihood of experiencing severe hypoglycemia [27]. In a separate study, it was discovered that severe hypoglycemia was commonly linked to the usage of glimepiride (24.2%) and neutral protamine Hagedorn-insulin and regular insulin “NPH/RI” (38.3%) [28]. Glimepiride stimulates insulin release by acting on ATPase-dependent potassium channels in pancreatic cells. It has been found in euglycemic and hyperglycemic clamp trials to improve both first- and second-phase insulin secretion [29].

Overall, studies suggest that several factors, including medication changes, lifestyle changes, and specific drug usage, contribute to the increased risk of severe hypoglycemia in patients with diabetes [30–32]. We conducted this narrative review to determine issues around the use of GLP-1 by nondiabetic obese individuals during Ramadan. Published papers were reviewed to provide a consolidated and comprehensive summary of existing research on the use of glucagon-like peptide-1 (GLP-1) receptor agonists during Ramadan fasting. This approach offers a well-structured, evidence-based narrative review, including expert opinions and recommendations, while ensuring the information was derived from multiple scientific studies and was readily accessible to a broad audience.

Several distinct combinations of the terms “nondiabetes,” “diabetes,” “type 2 diabetes,” “Ramadan,” “fasting,” “obesity,” “overweight,” “GLP,” “GLP-1,” “GLP-1RA,” “GLP agonist,” “GLP-1RA analog,” “Incretin mimetics,” and “antidiabetic drug” were used in several different combinations in several databases, including PubMed, Google Scholar, CiNAHL, and others. In addition, a broad range of publications were discovered, including practice and management guidelines published by a group of authors with a track record of publishing on this issue, as well as observational studies, randomized controlled trials (RCTs), systematic, nonsystematic, and topical reviews. 13 studies were examined for this evaluation, including four systematic reviews, three randomized trials, three sets of recommendations, and three other investigations (Table 1).

## 2. Safety Assessment of Glucose-Lowering Drugs and the Importance of Structured Education during Ramadan

Structured education involves informing diabetic patients on how to prevent hypoglycemia and other complications who fast during Ramadan [33]. To determine the percentage of individuals who experience hypoglycemia, changes in HbA1c levels, and the results of structured education for people taking glucose-lowering medications during Ramadan, the authors conducted a systematic review and meta-analysis of prior studies [34]. The findings demonstrated that a low incidence of hypoglycemia during Ramadan was related to glucose-lowering medications such as insulin, sulfonylureas, and metformin. The most recent generation of glucose-lowering medications has a lesser chance of resulting in hypoglycemia than sulphonylureas. Among sulphonylureas, gliclazide has been found to have one of the lowest rates of hypoglycemia. Compared to utilizing sulphonylureas, the results demonstrated that using DPP-4 inhibitors significantly lowers the incidence of hypoglycemia, with an odds ratio of 0.38 (95% confidence interval: 0.26 to 0.55, *p* value 0.00001). To conclude, newer medications are associated with a lower risk of hypoglycemia compared to sulfonylureas, and DPP-4 inhibitors are particularly effective in reducing this risk.

### 2.1. Efficacy and Safety of Newer Oral Hypoglycemic Agents in Patients with T2DM during Ramadan

In patients with type 2 diabetes during Ramadan, a systematic review and meta-analysis were conducted to assess the effectiveness and safety of more recent oral hypoglycemic medications [35, 36]. DPP-4i, GLP-1, and SGLT-2i diabetic medicines were each examined for their effects on HbA1c, weight, blood pressure, and hypoglycemia throughout the Ramadan fasting. DPP-4i was associated with a significant decline in HbA1c but not weight, according to a study that reviewed data from 20 studies in different countries. The findings demonstrated that, compared to sulfonylureas, DPP-4i was related to a decreased incidence of hypoglycemia and improved HbA1c and weight [35, 36]. In comparison to sulfonylureas, GLP-1 agonists and SGLT-2 inhibitors were linked to favorable outcomes, such as decreased HbA1c, weight, blood pressure, and a lower incidence of hypoglycemia, highlighting GLP-1's ability to lower the occurrence of hypoglycemia when fasting during the holy month of Ramadan [27]. However, it is significant to highlight that there was significant variability across the studies included in the meta-analysis, which emphasizes the significance of diabetic patients discussing the advantages and disadvantages of fasting during Ramadan with their healthcare professionals.

## 3. Impact of Ramadan Diurnal Intermittent Fasting on Hypoglycemic Events in Patients with Type 2 Diabetes

Another systematic review was conducted to assess how Ramadan's daily intermittent fasting affected individuals with type 2 diabetes who experienced hypoglycemia episodes [36]. Both observational studies and randomized controlled trials were included in the review. The outcomes evaluated were weight, systolic and diastolic blood pressure, hypoglycemic episodes, HbA1c (a marker of long-term glucose management), and hypoglycemia. The results showed a lower incidence of hypoglycemia during Ramadan's daily intermittent fasting compared to the pre-Ramadan period, especially in patients taking more recent diabetes medications, such as sodium-glucose transport-2 inhibitors, glucagon-like peptide-1 receptor agonists, and dipeptidyl peptidase-4 inhibitors (DPP-4i) (SGLT-2i) [25]. Patients with type 2 diabetes are therefore recommended to thoroughly examine the potential dangers and advantages of Ramadan daily intermittent fasting with their healthcare professionals.

### 3.1. Efficacy and Safety of the Current Hypoglycemic Agents in Patients with Diabetes during Ramadan Fasting

One study aimed to assess the efficiency and safety of hypoglycemic drugs in type 2 diabetics fasting during Ramadan [37]. This systematic review included randomized controlled trials and observational studies to look into the effects of various hypoglycemic medications on significant outcomes, such as weight, systolic and diastolic blood pressure, hypoglycemia, and HbA1c (a marker of long-term glucose control). The findings demonstrated that the use of new antidiabetic drugs, including sodium-glucose transport-2 inhibitor (SGLT-2i), glucagon-like peptide-1 receptor agonist, and dipeptidyl peptidase-4 inhibitor (DPP-4i), was associated with a decreased incidence of hypoglycemia when compared to pre-Ramadan and sulfonylurea therapies. Additionally, improvements in HbA1c, weight, and blood pressure were seen with GLP-1. Despite the differences between the included studies, particularly in reporting hypoglycemic events, this systematic review's findings concur with those of prior meta-analyses and support the notion that newer antidiabetic drugs can be helpful and safe during Ramadan fasting. However, studies suggest people with type 2 diabetes who want to fast during Ramadan to first see their doctor to discuss the benefits and dangers involved and, if necessary, modify their medication regimen.

### 3.2. Safety of Lixisenatide versus Sulfonylurea Added to Basal Insulin Treatment in People with Type 2 Diabetes Mellitus Who Elect to Fast during Ramadan

The safety of lixisenatide, a glucagon-like peptide-1 (GLP-1) receptor agonist, and sulfonylurea, when combined with basal insulin therapy, was examined in the LixiRam trial on people with type 2 diabetes who prefer to fast throughout Ramadan [38]. In terms of preserving glycemic control throughout Ramadan, the study found that lixisenatide was noninferior to sulfonylurea and was associated with a lower rate of hypoglycemia. Lixisenatide was also associated with greater body weight and HbA1c reduction than sulfonylurea (a marker of blood sugar management). The LixiRam trial suggests that lixisenatide may be a good sulfonylurea substitute for people with type 2 diabetes who opt to fast throughout Ramadan in terms of decreasing HbA1c, weight, and hypoglycemia [39]. Additionally, a post-hoc analysis of the LixiRam randomized trial's data was done [40] to evaluate the safety of a treatment plan using lixisenatide and basal insulin in people with type 2 diabetes mellitus (T2DM) while fasting during Ramadan was examined in the study. According to the data, a drop in HbA1c levels and a decreased incidence of hypoglycemia compared to baseline were indicators of improved glycemic control and safety of the treatment regimen during Ramadan fasting. These results suggest the potential advantages of GLP-1 receptor agonists, such as lixisenatide, when used with basal insulin to manage T2DM during the fasting month of Ramadan.

## 4. Strategies to Make Ramadan Fasting Safer for Type 2 Diabetics

The study examined many strategies that can be employed to make Ramadan fasting safer for those with type 2 diabetes [41]. Using a network meta-analysis that comprised randomized controlled trials and observational studies, a systematic review and network meta-analysis were used to investigate the effects of several treatments, such as medication adjustment, dietary changes, and lifestyle changes, on significant outcomes, including hypoglycemia, HbA1c (a marker of long-term glucose management), weight, systolic and diastolic blood pressure, and glycemic variability. The results showed that the usage of newer antidiabetic drugs, such as sodium-glucose transport-2 inhibitor (SGLT-2i), dipeptidyl peptidase-4 inhibitor (DPP-4i), and glucagon-like peptide-1 receptor agonist (GLP-1), has also been shown to help decrease blood pressure, weight, and HbA1c. In both RCTs (pooled relative risk: 0.56; 95% confidence interval: 0.44–0.72) and observational studies, the usage of DPP-4 (Dipeptidyl peptidase inhibitor-4) inhibitors was linked to a lower incidence of feeling hypoglycemia during Ramadan (pooled relative risk: 0.27; 0.09–0.75). The findings of the study, which are consistent with other systematic reviews and meta-analyses, suggest that type 2 diabetes patients may benefit from a combination of pharmaceutical changes—such as GLP-1—dietary changes, and lifestyle changes to lower their risk of hypoglycemia during the fasting month of Ramadan.

### 4.1. Efficacy and Safety of Liraglutide Compared to Sulphonylurea during Ramadan in Patients with Type 2 Diabetes (LIRA-Ramadan)

Participants with type 2 diabetes who were fasting during Ramadan participated in the LIRA-Ramadan study, which compared the safety and effectiveness of liraglutide, a glucagon-like peptide-1 (GLP-1) receptor agonist, and sulphonylurea, a commonly prescribed diabetic drug, with those of the two other treatments. According to documented data, patients on liraglutide had 2% fewer symptomatic hypoglycemic episodes than those taking sulphonylurea (11%, 18 patients). The study also found that liraglutide was associated with lower body weight and a more significant decline in HbA1c than sulphonylurea (a blood sugar control marker). Liraglutide may be a safe and practical choice for people with type 2 diabetes who want to fast throughout Ramadan, and it may be superior to sulphonylurea in terms of weight gain and hypoglycemia risk, according to the LIRA-Ramadan trial [42].

### 4.2. Comparison of GLP-1 Receptor Agonist Liraglutide to Sulphonylurea as an Add-On to Metformin in Patients with Established Type 2 Diabetes during Ramadan

Patients with type 2 diabetes who were fasting throughout Ramadan were given either liraglutide, a GLP-1 receptor agonist, or sulphonylurea as a supplementary medicine to metformin in the Treat 4 Ramadan investigation [43]. When glycemic control throughout Ramadan was evaluated in the trial, liraglutide was noninferior to sulphonylurea. In addition, a composite endpoint of hemoglobin A1c (HbA1c) of 7%, no weight gain, and no severe hypoglycemia at 12 weeks were achieved by more patients in the liraglutide group compared to the sulphonylurea group, albeit this did not reach statistical significance (odds ratio (OR) 4.08, 95% confidence interval (CI) 0.97, 17.22, *p*=0.06).

## 5. Canadian Statement on Fasting during Ramadan

According to a position statement the Canadian Diabetes Association (CDA) released on Ramadan fasting and diabetes, anyone with diabetes who is thinking about doing so should speak with their doctor to see if doing so is safe for them [44]. According to these guidelines, GLP-1 drugs are considered safe to use during Ramadan, and their use may even be advantageous due to their low risk of hypoglycemia. However, a pre-Ramadan assessment should be done one to three months before Ramadan, per Canadian Health enter recommendations for fasting during this holy month [45]. Additionally, dietary advice should be based on IDF-DAR and Diabetes Canada clinical practice standards, which should also be used to categorize individuals with diabetes who fall into very-high-risk special groups (such as geriatrics and pregnant women) [46].

### 5.1. South East London Area Prescribing Committee on Fasting during Ramadan

All people with diabetes considering fasting are advised by the South East London Area Prescribing Committee to schedule an appointment with their doctor two to three months before Ramadan or bring up the topic during their annual evaluation. These recommendations state that GLP-1 medications are acceptable during Ramadan, and patients should be encouraged to continue taking their medications throughout the holy month. However, to prevent gastrointestinal adverse effects, patients should be advised about the significance of dose titration at least four weeks before Ramadan. A risk assessment should also be performed, and patients and healthcare professionals should develop specialized management strategies for such patients [47].

### 5.2. IDF-DAR Practical Guidelines 2021

The 2021 IDF-DAR (International Diabetes Federation—Diabetes and Ramadan) Practical Guidelines' conclusions are based on the most recent research and the combined knowledge of experts in the field of diabetes care. The recommendations conclude that GLP-1 agonists can be used safely throughout Ramadan if taken according to the recommended schedule, and people with diabetes who use them are well monitored by their doctor. Additionally, the recommendations state that those with diabetes who take GLP-1 agonists should not stop taking them without consulting a healthcare professional, even if they are fasting throughout Ramadan.

### 5.3. Other Studies

Another study indicated that a structured teaching program dramatically reduced the number of hypoglycemic events among participants. Exercise, meal preparation, glucose monitoring, hypoglycemia, and scheduling and dosing of medications were only a few of the topics covered in the curriculum. As a result, five rather than nine hypoglycemic incidents occurred in this group. Contrarily, those who chose not to participate in the instructional program experienced an increase in hypoglycemic events from 9 to 36 (*p* 0.001). (2009) [48]. Studies have also shown the safety of utilizing the incretin-based treatment GLP-1 receptor agonist (liraglutide) during the fasting month of Ramadan. There were minimal disparities in age and gender distribution between the cohort of patients categorized as “unclassifiable” and the group comprising subjects diagnosed with type 2 diabetes. There was no significant increase in hypoglycemia occurrence after adding GLP-1 to an already effective antidiabetic treatment that included either sulfonylurea or insulin [49]. Another study has demonstrated that the use of incretin enhancers (DPP-4 inhibitors) and mimetics (GLP-1 agonists) alone, without combination with sulfonylurea, has a low risk of resulting in hypoglycemia [50].

## 6. Limitations

Further research is needed on using glucagon-like peptide-1 (GLP-1) receptor agonists during Ramadan in nondiabetics. Even though data from diabetic patients can be instructive, extrapolating these results must be done cautiously because nondiabetics may have a higher risk of hypoglycemia. To close this knowledge gap, there is a need for more thorough experimental research, such as case-control studies and randomized controlled trials that explicitly look at the risk of hypoglycemia associated with various GLP-1 receptor agonists in nondiabetic people during Ramadan.

These investigations would aid in determining which GLP-1 receptor agonist is more secure to employ in this population. In conclusion, research on GLP-1 receptor agonists in nondiabetic people during Ramadan still needs to be done. More data are urgently needed to understand better the risks and advantages of using these medications during this time. Therefore, the scientific community is urged to prioritize this field of study and carry out research that will instruct medical professionals and patients on how to use GLP-1 receptor agonists during Ramadan safely and effectively.

## 7. Conclusion

In conclusion, the body of evidence analyzed in this narrative review highlights the effectiveness and safety of glucagon-like peptide-1 (GLP-1) receptor agonists during Ramadan, especially in people with diabetes. These studies offer important information about the usage of GLP-1 therapies and reassurance about their low risk of hypoglycemia in comparison to other antidiabetic drugs. The results also highlight the value of patient education, particularly for those who had a history of hypoglycemia before Ramadan as they are more vulnerable during the fasting time. To guarantee the safe and efficient use of GLP-1 medications, close monitoring and dose titration, preferably at least four weeks before Ramadan, are essential procedures.

However, it is important to note that while these studies have contributed significantly to our understanding of GLP-1 therapy during Ramadan, more research is needed, particularly in the context of nondiabetic individuals who fast during this religious observance. As of now, there is a dearth of studies focused specifically on the use of GLP-1 medications in nondiabetic patients during Ramadan. To fully comprehend the potential benefits and limitations of GLP-1 therapy for this population, further investigations are essential. In conclusion, the existing evidence strongly supports the safe and effective use of GLP-1 drugs during Ramadan, with ongoing research needed to expand our knowledge, particularly in nondiabetic individuals who undertake fasting during this sacred month.

## Figures and Tables

**Table 1:** Studies included in the narrative review.

Study design	Studies reviewed
Systematic reviews	(1) Safety Assessment of Glucose-Lowering Drugs and Importance of Structured Education during Ramadan: A Systematic Review and Meta-Analysis
	(2) Efficacy and Safety of Newer Oral Hypoglycemic Agents in Patients with T2DM during Ramadan: A Systematic Review and Meta-Analysis
	(3) Impact of Ramadan Diurnal Intermittent Fasting on Hypoglycemic Events in Patients with Type 2 Diabetes: A Systematic Review of Randomized Controlled Trials and Observational Studies
	(4) A Systematic Review on Efficacy and Safety of the Current Hypoglycemic Agents in Patients with Diabetes during Ramadan Fasting

Randomized trials	(5) Safety of Lixisenatide versus Sulfonylurea Added to Basal Insulin Treatment in People with Type 2 Diabetes Mellitus Who Elect to Fast during Ramadan (LixiRam): An International, Randomized, Open-Label Trial
	(6) Efficacy and Safety of Liraglutide Compared to Sulphonylurea during Ramadan in Patients with Type 2 Diabetes (LIRA-Ramadan): A Randomized Trial
	(7) Treat 4 Ramadan Trial A Randomized Controlled Trial Comparing the GLP-1 Receptor Agonist Liraglutide to Sulphonylurea as an Add-On to Metformin in Patients with Established Type 2 Diabetes during Ramadan: The Treat 4 Ramadan Trial

Guidelines	(8) Canadian Statement on Fasting during Ramadan
	(9) South East London Area Prescribing Committee on fasting during Ramadan
	(10) IDF-DAR practical guidelines 2021

Other studies	(11) Ramadan Education and Awareness in Diabetes (READ) program for Muslims with type 2 diabetes who fast during Ramadan
	(12) Safety and Efficacy of Liraglutide as an Add-On Therapy to Preexisting Antidiabetic Regimens during Ramadan, a Prospective Observational Trial
	(13) Religious Fasting, Ramadan, and Hypoglycemia in People with Diabetes
